# Rasmussen’s aneurysm

**DOI:** 10.36416/1806-3756/e20240286

**Published:** 2024-12-17

**Authors:** Edson Marchiori, Bruno Hochhegger, Gláucia Zanetti

**Affiliations:** 1. Universidade Federal do Rio de Janeiro, Rio de Janeiro (RJ) Brasil.; 2. University of Florida, Gainesville (FL) USA.

**Figure 1:**
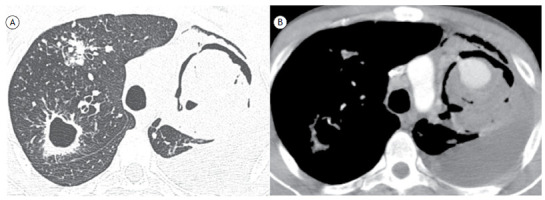
In A, CT scan of the chest with lung window settings at the level of the upper lobes, showing nodules and a thick-walled cavity in the right upper lobe. On the left, note a large cavitary lesion and an intracavitary mass, with air interposed between the mass and the cavity wall (the air crescent sign). In B, CT scan of the chest with mediastinal window settings after administration of intravenous contrast, showing intense enhancement of the intracavitary mass, consistent with a diagnosis of aneurysm formation (Rasmussen’s aneurysm).

A 39-year-old man presented with a three-month history of cough and hemoptysis, as well as anorexia and weight loss (12 kg). A CT scan of the chest showed nodules and a thick-walled cavity in the right lung, as well as a large cavitary lesion on the left, containing a round mass surrounded by a halo of air (the air crescent sign). The mass showed intense enhancement after administration of iodinated contrast (Figure 1). The final diagnosis was active tuberculosis with a Rasmussen aneurysm (RA) on the left. 

In patients with tuberculosis, hemoptysis is frequently caused by erosion of a bronchial artery or a pulmonary artery branch. Common causes of hemoptysis include bronchiectasis, aspergilloma, reactivation of tuberculosis, lung scar carcinoma, broncholithiasis, and RA. Contrast-enhanced chest CT and bronchoscopy are the methods of choice for the evaluation of pulmonary hemorrhage.^1^ ^,^ ^2^

The finding of a nodule or mass in a lung cavity has important diagnostic and therapeutic implications. Although aspergilloma is the most common cause of an intracavitary nodule, the differential diagnosis should include neoplasms (particularly bronchial carcinoma), (recovery phase of) angioinvasive aspergillosis, RA, and clots. The air crescent sign is commonly seen in patients with intracavitary nodules of any etiology. The air crescent sign is a crescent- or half-moon-shaped collection of air in the periphery of an intracavitary nodule or mass, separating the nodule or mass from the cavity wall.^1^ ^,^ ^3^ ^,^ ^4^

A change in the position of the nodule in the cavity when patient position is changed, especially during CT examinations in the supine and prone positions, can be useful in the differential diagnosis. It is extremely important to determine whether the nodule is free or attached to the cavity wall because, unlike a fungus ball or a clot, RA and cavitary lung cancer present as masses that are fixed to the cavity wall; that is, they do not move when patient position is changed. Intense contrast enhancement of the nodule is seen on CT scans of patients with RA and is useful in differentiating between aspergilloma and RA.^1^ ^,^ ^3^ ^,^ ^4^

In conclusion, RA should be included in the differential diagnosis of hemoptysis in tuberculosis patients presenting with the air crescent sign. Contrast-enhanced CT plays an important role in the evaluation of such patients. 
